# Bis(azido-κ*N*)bis­[4-(dimethyl­amino)­pyridine-κ*N*]zinc

**DOI:** 10.1107/S1600536813004686

**Published:** 2013-02-23

**Authors:** Fatiha Guenifa, Nasreddine Hadjadj, Ouahida Zeghouan, Lamia Bendjeddou, Hocine Merazig

**Affiliations:** a Unité de Recherche de Chimie de l’Environnement et Moléculaire Structurale (CHEMS), Faculté des Sciences Exactes, Campus Chaabet Ersas, Université Constantine I, 25000 Constantine, Algeria

## Abstract

In the title complex, [Zn(N_3_)_2_(C_7_H_10_N_2_)_2_], the Zn^II^ atom is coordinated by two N atoms from two 4-(dimethyl­amino)­pyridine (DMAP) ligands and by two N atoms from two azide anions in a distorted tetra­hedral coordination geometry. In the crystal, weak C—H⋯N hydrogen bonds between the DMAP and azide ligands link these discrete complex mol­ecules into a three-dimensional supra­molecular network.

## Related literature

For the property of complexes with a dimethyl­amino­pyridine ligand, see: Araki *et al.* (2005 ▶). For weak hydrogen-bonding modes, see: Bernstein *et al.* (1995 ▶). For related compounds, see: Fu (2000 ▶); Tyrra *et al.* (2003 ▶).
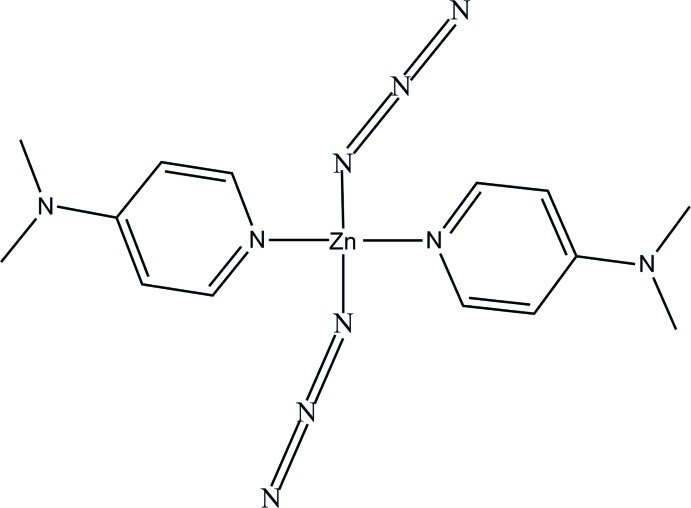


## Experimental

### 

#### Crystal data

[Zn(N_3_)_2_(C_7_H_10_N_2_)_2_]
*M* *_r_* = 393.79
Monoclinic, 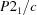

*a* = 14.819 (5) Å
*b* = 9.610 (5) Å
*c* = 14.555 (5) Å
β = 118.158 (5)°
*V* = 1827.5 (13) Å^3^
*Z* = 4
Mo *K*α radiation
μ = 1.36 mm^−1^
*T* = 293 K
0.3 × 0.2 × 0.2 mm

#### Data collection

Nonius KappaCCD diffractometer
15768 measured reflections
4302 independent reflections
3323 reflections with *I* > 2σ(*I*)
*R*_int_ = 0.019

#### Refinement

*R*[*F* ^2^ > 2σ(*F* ^2^)] = 0.033
*wR*(*F* ^2^) = 0.098
*S* = 1.03
4302 reflections
230 parameters
H-atom parameters constrained
Δρ_max_ = 0.31 e Å^−3^
Δρ_min_ = −0.45 e Å^−3^

### 

Data collection: *KappaCCD Reference Manual* (Nonius, 1998 ▶); cell refinement: *DENZO* and *SCALEPACK* (Otwinowski & Minor, 1997 ▶); data reduction: *DENZO* and *SCALEPACK* ▶; program(s) used to solve structure: *SIR2002* (Burla *et al.*, 2003 ▶); program(s) used to refine structure: *SHELXL97* (Sheldrick, 2008 ▶); molecular graphics: *ORTEP-3 for Windows* (Farrugia, 2012 ▶); software used to prepare material for publication: *WinGX* (Farrugia, 2012 ▶), *Mercury* (Macrae *et al.*, 2006 ▶) and *POVRay* (Persistence of Vision Team, 2004 ▶).

## Supplementary Material

Crystal structure: contains datablock(s) global, I. DOI: 10.1107/S1600536813004686/xu5676sup1.cif

Structure factors: contains datablock(s) I. DOI: 10.1107/S1600536813004686/xu5676Isup2.hkl

Additional supplementary materials:  crystallographic information; 3D view; checkCIF report

## Figures and Tables

**Table 1:** Selected bond lengths (Å)

Zn—N1*A*	2.031 (2)
Zn—N1*B*	2.018 (2)
Zn—N3	1.935 (3)
Zn—N6	1.969 (3)

**Table 2:** Hydrogen-bond geometry (Å, °)

*D*—H⋯*A*	*D*—H	H⋯*A*	*D*⋯*A*	*D*—H⋯*A*
C1*A*—H1*A*1⋯N8^i^	0.96	2.56	3.484 (4)	163
C5*A*—H5*A*⋯N8^ii^	0.93	2.59	3.332 (4)	137
C7*B*—H7*B*⋯N3^iii^	0.93	2.58	3.367 (4)	143
C7*B*—H7*B*⋯N4^iii^	0.93	2.59	3.382 (4)	143

Symmetry codes: (i) 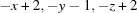; (ii) 
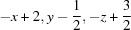
; (iii) 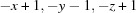.

**Fig. 1.**
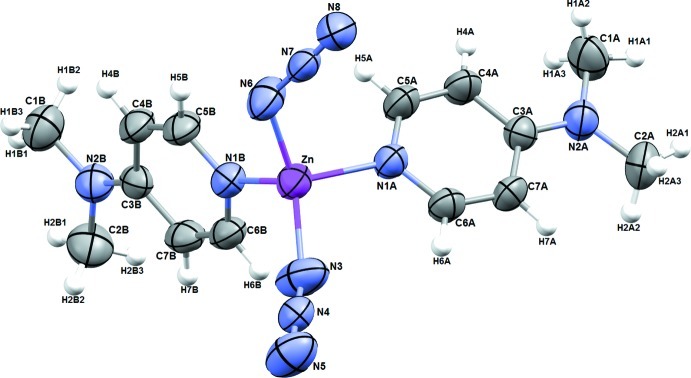
The asymmetric unit of the title structure with the atom numbering scheme. Displacement ellipsoids are drawn at the 50% probability level and H atoms are represented as small spheres of arbitrary radii.

**Fig. 2.**
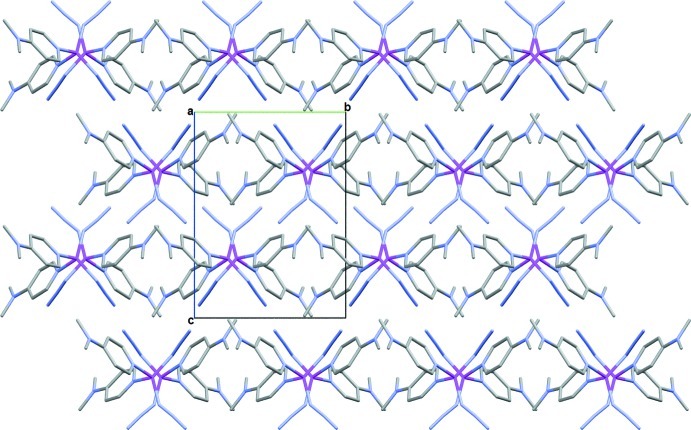
view of the crystal structure of title compound, showing double layers along the b axis. Hydrogen atoms are omitted for clarity.

**Fig. 3.**
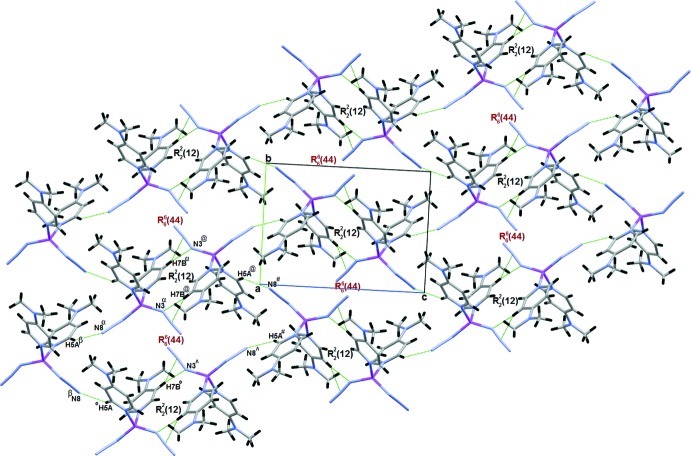
Part of the crystal structure, showing the aggregation of R22(12) and R66(44) hydrogen-bonding motifs. [Symmetry codes: (α) –x+1/2, y - 1/2, -z; (β) –x, -y, -z - 1; (@) –x+1/2, y - 1/2, -z; (#) x, y - 1, z; (°) x - 1/2, -y - 1/2, z - 1; (^) –x+1/2, y - 3/2, -z].

## supplementary crystallographic information

### Comment

Pyridine derivatives are an important class of ligand for constructing metal-organic frameworks. 4-Dimethylaminopyridine (DMAP) has good coordination ability, but there are few reports on its complexes (Tyrra *et al.*, 2003) except a lot of reports on its nucleophilic properties (Fu *et al.*, 2000). The DMAP complexes which exhibit luminescence properties were reported (Araki *et al.*, 2005). In our systematic studies on transition metal complexes with the pyridine derivatives and other ligands, the title compound [Zn(C_7_H_10_N_2_)_2_(N_3_)_2_] was prepared and its X-ray structure is presented here.

The title complex (I), is a mononuclear Zn(II) complex, consisting of two 4-dimethylaminopyridine (DMAP) ligands and two azide anions (Figure 1), all ligands coordinating in a monodentate manner. The title compound exhibits a distorted tetrahedral coordination involving two N atoms from two 4-dimethylaminopyridine (DMAP) ligands at 2.031 (2), 2.018 (2) Å and two N atoms from two azide anions at 1.934 (3), 1.968 (2) Å, all coordinated in a monodentate fashion (Figure 1). The bond angles around Zn atom vary from 103.45 (13) to 127.06 (11)°.

The crystal structure can be described as double layers which stack along the *b* axis, at c = 1/4 and 3/4, where each layer is formed of pairs of monomers (Figure 2). In the structure of (I), mononuclear units are held together with weak intermolecular C—H···N hydrogen bonds between the 4-dimethylaminopyridine and the azide, forming an alternating centrosymmetric rings in two-dimensional network which can be described by the graph-set motif *R*^2^_2_(12) and *R*^6^_6_(44) (Bernstein *et al.*, 1995) (Figure 3). The combination of the four intermolecular C—H···N hydrogen bonds generates a three-dimensional network.

### Experimental

The title compound is prepared by reaction of methanolic solution containing Zn(CH_3_COO)_2_.2H_2_O, NaN_3_ and 4-dimethylaminopyridine in a 1:1:1 stoichiometric ratio. The solution was maintained in 293 K under agitation during one hour. Colorless crystals were appeared by evaporation of the solution at room temperature over the course of a few days.

### Refinement

The H atoms were placed at calculated positions with C—H = 0.93 and 0.96 Å, for aromatic and methyl H atoms, respectively, and refined in riding mode with *U*_iso_(H) = 1.2*U*_eq_(C) for aromatic H atoms and 1.5*U*_eq_(C) for methyl H atoms.

### Crystal data

**Table:**

[Zn(N_3_)_2_(C_7_H_10_N_2_)_2_]	*F*(000) = 816
*M**_r_* = 393.79	*D*_x_ = 1.431 Mg m^−^^3^
Monoclinic, *P*2_1_/*c*	Mo *K*α radiation, λ = 0.71073 Å
Hall symbol: -P 2ybc	Cell parameters from 4302 reflections
*a* = 14.819 (5) Å	θ = 2.7–27.8°
*b* = 9.610 (5) Å	µ = 1.36 mm^−^^1^
*c* = 14.555 (5) Å	*T* = 293 K
β = 118.158 (5)°	Prism, colourless
*V* = 1827.5 (13) Å^3^	0.3 × 0.2 × 0.2 mm
*Z* = 4	

### Data collection

**Table:**

Nonius KappaCCD diffractometer	3323 reflections with *I* > 2σ(*I*)
Radiation source: fine-focus sealed tube	*R*_int_ = 0.019
Graphite monochromator	θ_max_ = 27.8°, θ_min_ = 2.7°
φ scans	*h* = −18→19
15768 measured reflections	*k* = −12→12
4302 independent reflections	*l* = −19→17

### Refinement

**Table:**

Refinement on *F*^2^	0 restraints
Least-squares matrix: full	H-atom parameters constrained
*R*[*F*^2^ > 2σ(*F*^2^)] = 0.033	*w* = 1/[σ^2^(*F*_o_^2^) + (0.0512*P*)^2^ + 0.563*P*] where *P* = (*F*_o_^2^ + 2*F*_c_^2^)/3
*wR*(*F*^2^) = 0.098	(Δ/σ)_max_ = 0.006
*S* = 1.03	Δρ_max_ = 0.31 e Å^−^^3^
4302 reflections	Δρ_min_ = −0.45 e Å^−^^3^
230 parameters	

### Special details

**Table:**

Geometry. Bond distances, angles *etc*. have been calculated using the rounded fractional coordinates. All su's are estimated from the variances of the (full) variance-covariance matrix. The cell e.s.d.'s are taken into account in the estimation of distances, angles and torsion angles
Refinement. Refinement of *F*^2^ against ALL reflections. The weighted *R*-factor *wR* and goodness of fit *S* are based on *F*^2^, conventional *R*-factors *R* are based on *F*, with *F* set to zero for negative *F*^2^. The threshold expression of *F*^2^ > σ(*F*^2^) is used only for calculating *R*-factors(gt) *etc*. and is not relevant to the choice of reflections for refinement. *R*-factors based on *F*^2^ are statistically about twice as large as those based on *F*, and *R*- factors based on ALL data will be even larger.

### Fractional atomic coordinates and isotropic or equivalent isotropic displacement parameters (Å^2^)

**Table:**

	*x*	*y*	*z*	*U*_iso_*/*U*_eq_	
Zn	0.71191 (2)	−0.27848 (3)	0.61121 (2)	0.0523 (1)	
N1A	0.77350 (13)	−0.40503 (19)	0.73790 (13)	0.0500 (5)	
N1B	0.69230 (13)	−0.40145 (19)	0.49076 (13)	0.0504 (5)	
N2A	0.89709 (15)	−0.6937 (2)	0.98096 (15)	0.0595 (6)	
N2B	0.64450 (14)	−0.6645 (2)	0.24262 (14)	0.0571 (6)	
N3	0.57850 (19)	−0.2334 (3)	0.5965 (2)	0.0873 (10)	
N4	0.52367 (14)	−0.1422 (2)	0.59095 (14)	0.0545 (6)	
N5	0.4650 (2)	−0.0614 (3)	0.5847 (2)	0.0865 (10)	
N6	0.81913 (18)	−0.1468 (2)	0.62440 (17)	0.0693 (8)	
N7	0.87832 (14)	−0.0916 (2)	0.70190 (16)	0.0531 (6)	
N8	0.93719 (16)	−0.0315 (3)	0.77445 (17)	0.0734 (8)	
C1A	0.9886 (2)	−0.7725 (3)	0.9999 (2)	0.0752 (10)	
C1B	0.7054 (2)	−0.6558 (3)	0.1887 (2)	0.0721 (9)	
C2A	0.8539 (2)	−0.7179 (3)	1.05133 (19)	0.0660 (9)	
C2B	0.5557 (2)	−0.7569 (3)	0.1963 (2)	0.0751 (9)	
C3A	0.85764 (15)	−0.5982 (2)	0.90434 (15)	0.0475 (6)	
C3B	0.66005 (15)	−0.5783 (2)	0.32121 (15)	0.0470 (6)	
C4A	0.90324 (17)	−0.5695 (3)	0.84037 (18)	0.0570 (7)	
C4B	0.73862 (17)	−0.4775 (2)	0.36129 (17)	0.0557 (7)	
C5A	0.85983 (17)	−0.4761 (3)	0.76167 (18)	0.0583 (8)	
C5B	0.75054 (17)	−0.3949 (3)	0.44220 (17)	0.0565 (7)	
C6A	0.73042 (16)	−0.4308 (2)	0.80006 (16)	0.0515 (7)	
C6B	0.61798 (16)	−0.4982 (2)	0.45299 (18)	0.0535 (7)	
C7A	0.76869 (16)	−0.5216 (2)	0.88114 (16)	0.0511 (7)	
C7B	0.59908 (16)	−0.5840 (2)	0.37233 (18)	0.0541 (7)	
H1A2	0.97840	−0.81574	0.93625	0.1128*	
H1A1	1.00122	−0.84274	1.05144	0.1128*	
H1A3	1.04621	−0.71070	1.02438	0.1128*	
H1B1	0.68314	−0.57774	0.14203	0.1081*	
H4A	0.96322	−0.61489	0.85225	0.0684*	
H4B	0.78234	−0.46748	0.33231	0.0668*	
H1B2	0.69720	−0.73974	0.14972	0.1081*	
H5A	0.89213	−0.45992	0.72125	0.0699*	
H5B	0.80265	−0.32898	0.46580	0.0677*	
H1B3	0.77621	−0.64426	0.23863	0.1081*	
H6A	0.67072	−0.38324	0.78634	0.0618*	
H6B	0.57653	−0.50671	0.48472	0.0642*	
H2A1	0.86978	−0.64053	1.09819	0.0990*	
H7A	0.73584	−0.53312	0.92153	0.0613*	
H7B	0.54548	−0.64750	0.35014	0.0650*	
H2A2	0.88255	−0.80139	1.09057	0.0990*	
H2A3	0.78097	−0.72765	1.01151	0.0990*	
H2B1	0.55497	−0.81535	0.24940	0.1128*	
H2B2	0.55951	−0.81363	0.14396	0.1128*	
H2B3	0.49417	−0.70223	0.16501	0.1128*	

### Atomic displacement parameters (Å^2^)

**Table:**

	*U*^11^	*U*^22^	*U*^33^	*U*^12^	*U*^13^	*U*^23^
Zn	0.0535 (2)	0.0544 (2)	0.0550 (2)	0.0003 (1)	0.0306 (1)	0.0062 (1)
N1A	0.0505 (9)	0.0562 (10)	0.0507 (9)	0.0035 (8)	0.0299 (8)	0.0048 (8)
N1B	0.0488 (9)	0.0563 (10)	0.0493 (9)	−0.0028 (8)	0.0259 (8)	0.0053 (8)
N2A	0.0636 (11)	0.0677 (12)	0.0524 (10)	0.0111 (9)	0.0316 (9)	0.0107 (9)
N2B	0.0592 (11)	0.0567 (10)	0.0543 (10)	−0.0101 (9)	0.0260 (9)	−0.0023 (9)
N3	0.0676 (14)	0.0791 (15)	0.133 (2)	0.0182 (12)	0.0619 (16)	0.0337 (15)
N4	0.0539 (10)	0.0610 (11)	0.0555 (10)	−0.0035 (9)	0.0315 (9)	0.0063 (9)
N5	0.0932 (17)	0.0744 (15)	0.119 (2)	0.0149 (14)	0.0724 (16)	0.0096 (14)
N6	0.0831 (14)	0.0720 (14)	0.0663 (13)	−0.0223 (12)	0.0464 (12)	−0.0047 (11)
N7	0.0517 (10)	0.0619 (11)	0.0593 (11)	0.0050 (9)	0.0374 (10)	0.0141 (9)
N8	0.0582 (12)	0.1026 (17)	0.0617 (12)	−0.0082 (12)	0.0301 (10)	0.0011 (12)
C1A	0.0760 (17)	0.0805 (17)	0.0680 (16)	0.0282 (14)	0.0331 (14)	0.0180 (13)
C1B	0.0857 (17)	0.0749 (17)	0.0660 (15)	−0.0100 (14)	0.0442 (14)	−0.0078 (13)
C2A	0.0845 (17)	0.0680 (15)	0.0526 (13)	0.0008 (13)	0.0382 (13)	0.0065 (11)
C2B	0.0756 (17)	0.0686 (15)	0.0731 (17)	−0.0221 (13)	0.0284 (14)	−0.0147 (13)
C3A	0.0468 (10)	0.0538 (11)	0.0436 (10)	0.0001 (9)	0.0227 (9)	−0.0029 (9)
C3B	0.0429 (10)	0.0474 (11)	0.0465 (10)	−0.0017 (8)	0.0176 (9)	0.0104 (8)
C4A	0.0506 (11)	0.0679 (14)	0.0637 (13)	0.0142 (10)	0.0362 (10)	0.0113 (11)
C4B	0.0547 (12)	0.0661 (13)	0.0554 (12)	−0.0146 (11)	0.0335 (10)	−0.0008 (10)
C5A	0.0559 (12)	0.0734 (14)	0.0602 (13)	0.0069 (11)	0.0395 (11)	0.0087 (11)
C5B	0.0521 (11)	0.0654 (13)	0.0575 (12)	−0.0201 (10)	0.0305 (10)	−0.0037 (10)
C6A	0.0484 (11)	0.0582 (12)	0.0559 (12)	0.0064 (9)	0.0313 (10)	0.0020 (10)
C6B	0.0462 (10)	0.0581 (12)	0.0643 (13)	−0.0008 (9)	0.0327 (10)	0.0113 (10)
C7A	0.0530 (11)	0.0616 (13)	0.0503 (11)	0.0022 (10)	0.0340 (10)	−0.0006 (9)
C7B	0.0435 (10)	0.0530 (12)	0.0670 (13)	−0.0089 (9)	0.0270 (10)	0.0053 (10)

### Geometric parameters (Å, º)

**Table:**

Zn—N1A	2.031 (2)	C4B—C5B	1.361 (3)
Zn—N1B	2.018 (2)	C6A—C7A	1.358 (3)
Zn—N3	1.935 (3)	C6B—C7B	1.351 (3)
Zn—N6	1.969 (3)	C1A—H1A2	0.9600
N1A—C5A	1.344 (4)	C1A—H1A1	0.9600
N1A—C6A	1.354 (3)	C1A—H1A3	0.9600
N1B—C5B	1.351 (3)	C1B—H1B1	0.9600
N1B—C6B	1.345 (3)	C1B—H1B2	0.9600
N2A—C1A	1.461 (4)	C1B—H1B3	0.9600
N2A—C2A	1.460 (4)	C2A—H2A1	0.9600
N2A—C3A	1.346 (3)	C2A—H2A2	0.9600
N2B—C1B	1.451 (4)	C2A—H2A3	0.9600
N2B—C2B	1.462 (4)	C2B—H2B1	0.9600
N2B—C3B	1.341 (3)	C2B—H2B2	0.9600
N3—N4	1.172 (4)	C2B—H2B3	0.9600
N4—N5	1.137 (4)	C4A—H4A	0.9300
N6—N7	1.179 (3)	C4B—H4B	0.9300
N7—N8	1.158 (3)	C5A—H5A	0.9300
C3A—C4A	1.412 (4)	C5B—H5B	0.9300
C3A—C7A	1.405 (3)	C6A—H6A	0.9300
C3B—C4B	1.412 (3)	C6B—H6B	0.9300
C3B—C7B	1.417 (4)	C7A—H7A	0.9300
C4A—C5A	1.355 (4)	C7B—H7B	0.9300
			
Zn···H1B1^i^	3.5000	C7B···H2B3	2.9000
N1A···N1B	3.210 (3)	C7B···H2B1	2.7300
N1A···N3	3.113 (4)	C7B···H2A3^xi^	3.0800
N1A···N6	3.225 (3)	H1A2···C4A	2.7100
N1B···N1A	3.210 (3)	H1A2···H4A	2.2400
N1B···N3	3.205 (4)	H1A1···H2A2	2.1300
N1B···N6	3.139 (3)	H1A1···N8^vii^	2.5600
N3···N1A	3.113 (4)	H1A3···C4A	2.8500
N3···N1B	3.205 (4)	H1A3···H4A	2.3900
N3···C6A	3.339 (4)	H1A3···H5B^ix^	2.4500
N3···C7B^ii^	3.367 (4)	H1B1···C4B	3.0500
N4···N5^iii^	3.286 (4)	H1B1···Zn^vi^	3.5000
N4···C7B^ii^	3.382 (4)	H4A···C1A	2.5100
N5···C2B^i^	3.427 (4)	H4A···H1A2	2.2400
N5···N4^iii^	3.286 (4)	H4A···H1A3	2.3900
N6···N1B	3.139 (3)	H4A···N7^ix^	2.8200
N6···C5B	3.348 (4)	H4B···C1B	2.5900
N6···N1A	3.225 (3)	H4B···H1B3	2.1500
N7···C1B^i^	3.434 (4)	H4B···N7^vi^	2.9100
N8···C5A^iv^	3.332 (4)	H4B···N8^vi^	2.7900
N3···H6A	2.8300	H1B2···H2B2	2.1200
N3···H7B^ii^	2.5800	H5A···N8^ix^	2.5900
N4···H7B^ii^	2.5900	H5B···N6	2.8100
N5···H2B1^ii^	2.8300	H5B···C1A^iv^	2.9400
N5···H7A^v^	2.9500	H5B···H1A3^iv^	2.4500
N5···H2B3^i^	2.7400	H1B3···C4B	2.6500
N6···H2A1^vi^	2.9300	H1B3···H4B	2.1500
N6···H5B	2.8100	H1B3···N7^vi^	2.9100
N7···H4A^iv^	2.8200	H1B3···N8^vi^	2.7600
N7···H1B3^i^	2.9100	H6A···N3	2.8300
N7···H2A1^vi^	2.6600	H6B···H6B^ii^	2.5100
N7···H4B^i^	2.9100	H2A1···C7A	3.0100
N8···H5A^iv^	2.5900	H2A1···N6^i^	2.9300
N8···H1A1^vii^	2.5600	H2A1···N7^i^	2.6600
N8···H2A2^vii^	2.9400	H2A1···N8^i^	2.8100
N8···H4B^i^	2.7900	H7A···C2A	2.5800
N8···H1B3^i^	2.7600	H7A···H2A3	2.2000
N8···H2A1^vi^	2.8100	H7A···N5^xii^	2.9500
C1B···N7^vi^	3.434 (4)	H7B···C2B	2.5400
C2B···N5^vi^	3.427 (4)	H7B···H2B1	2.2300
C2B···C6B^viii^	3.383 (4)	H7B···H2B3	2.4900
C5A···N8^ix^	3.332 (4)	H7B···N3^ii^	2.5800
C6B···C2B^x^	3.383 (4)	H7B···N4^ii^	2.5900
C7B···N3^ii^	3.367 (4)	H2A2···H1A1	2.1300
C7B···N4^ii^	3.382 (4)	H2A2···N8^vii^	2.9400
C1A···H5B^ix^	2.9400	H2A3···C7A	2.6900
C1A···H4A	2.5100	H2A3···H7A	2.2000
C1B···H4B	2.5900	H2A3···C7B^xiii^	3.0800
C2A···H7A	2.5800	H2B1···C7B	2.7300
C2B···H7B	2.5400	H2B1···H7B	2.2300
C4A···H1A2	2.7100	H2B1···N5^ii^	2.8300
C4A···H1A3	2.8500	H2B2···H1B2	2.1200
C4B···H1B3	2.6500	H2B2···C6B^viii^	2.9200
C4B···H1B1	3.0500	H2B3···C7B	2.9000
C6B···H2B2^x^	2.9200	H2B3···H7B	2.4900
C7A···H2A1	3.0100	H2B3···N5^vi^	2.7400
C7A···H2A3	2.6900		
			
N1A—Zn—N1B	104.89 (7)	N2A—C1A—H1A3	109.00
N1A—Zn—N3	103.45 (10)	H1A2—C1A—H1A1	109.00
N1A—Zn—N6	107.47 (9)	H1A2—C1A—H1A3	109.00
N1B—Zn—N3	108.32 (10)	H1A1—C1A—H1A3	110.00
N1B—Zn—N6	103.88 (9)	N2B—C1B—H1B1	110.00
N3—Zn—N6	127.05 (11)	N2B—C1B—H1B2	109.00
Zn—N1A—C5A	120.24 (16)	N2B—C1B—H1B3	109.00
Zn—N1A—C6A	124.39 (16)	H1B1—C1B—H1B2	109.00
C5A—N1A—C6A	115.28 (19)	H1B1—C1B—H1B3	109.00
Zn—N1B—C5B	124.17 (16)	H1B2—C1B—H1B3	109.00
Zn—N1B—C6B	120.96 (16)	N2A—C2A—H2A1	109.00
C5B—N1B—C6B	114.9 (2)	N2A—C2A—H2A2	109.00
C1A—N2A—C2A	117.4 (2)	N2A—C2A—H2A3	109.00
C1A—N2A—C3A	120.7 (2)	H2A1—C2A—H2A2	109.00
C2A—N2A—C3A	121.8 (2)	H2A1—C2A—H2A3	109.00
C1B—N2B—C2B	116.1 (2)	H2A2—C2A—H2A3	109.00
C1B—N2B—C3B	121.7 (2)	N2B—C2B—H2B1	110.00
C2B—N2B—C3B	121.6 (2)	N2B—C2B—H2B2	109.00
Zn—N3—N4	144.5 (2)	N2B—C2B—H2B3	109.00
N3—N4—N5	174.7 (3)	H2B1—C2B—H2B2	110.00
Zn—N6—N7	125.8 (2)	H2B1—C2B—H2B3	109.00
N6—N7—N8	176.0 (3)	H2B2—C2B—H2B3	109.00
N2A—C3A—C4A	121.6 (2)	C3A—C4A—H4A	120.00
N2A—C3A—C7A	123.2 (2)	C5A—C4A—H4A	120.00
C4A—C3A—C7A	115.21 (19)	C3B—C4B—H4B	120.00
N2B—C3B—C4B	123.5 (2)	C5B—C4B—H4B	120.00
N2B—C3B—C7B	121.9 (2)	N1A—C5A—H5A	118.00
C4B—C3B—C7B	114.55 (19)	C4A—C5A—H5A	118.00
C3A—C4A—C5A	120.2 (2)	N1B—C5B—H5B	118.00
C3B—C4B—C5B	120.2 (2)	C4B—C5B—H5B	118.00
N1A—C5A—C4A	124.7 (2)	N1A—C6A—H6A	118.00
N1B—C5B—C4B	124.9 (2)	C7A—C6A—H6A	118.00
N1A—C6A—C7A	124.3 (2)	N1B—C6B—H6B	118.00
N1B—C6B—C7B	124.8 (2)	C7B—C6B—H6B	118.00
C3A—C7A—C6A	120.4 (2)	C3A—C7A—H7A	120.00
C3B—C7B—C6B	120.7 (2)	C6A—C7A—H7A	120.00
N2A—C1A—H1A2	109.00	C3B—C7B—H7B	120.00
N2A—C1A—H1A1	109.00	C6B—C7B—H7B	120.00
			
N1B—Zn—N1A—C5A	55.4 (2)	C6B—N1B—C5B—C4B	−0.3 (3)
N1B—Zn—N1A—C6A	−120.80 (17)	Zn—N1B—C6B—C7B	179.51 (18)
N3—Zn—N1A—C5A	168.85 (19)	C5B—N1B—C6B—C7B	−0.6 (3)
N3—Zn—N1A—C6A	−7.35 (19)	C1A—N2A—C3A—C4A	−0.6 (3)
N6—Zn—N1A—C5A	−54.7 (2)	C1A—N2A—C3A—C7A	178.6 (2)
N6—Zn—N1A—C6A	129.09 (17)	C2A—N2A—C3A—C4A	176.3 (2)
N1A—Zn—N1B—C5B	−107.05 (19)	C2A—N2A—C3A—C7A	−4.5 (3)
N1A—Zn—N1B—C6B	72.84 (18)	C1B—N2B—C3B—C4B	−3.1 (3)
N3—Zn—N1B—C5B	143.0 (2)	C1B—N2B—C3B—C7B	178.3 (2)
N3—Zn—N1B—C6B	−37.13 (19)	C2B—N2B—C3B—C4B	−173.3 (2)
N6—Zn—N1B—C5B	5.6 (2)	C2B—N2B—C3B—C7B	8.1 (3)
N6—Zn—N1B—C6B	−174.48 (17)	N2A—C3A—C4A—C5A	177.8 (2)
N1A—Zn—N3—N4	124.7 (4)	C7A—C3A—C4A—C5A	−1.5 (3)
N1B—Zn—N3—N4	−124.4 (3)	N2A—C3A—C7A—C6A	−177.3 (2)
N6—Zn—N3—N4	0.1 (4)	C4A—C3A—C7A—C6A	2.0 (3)
N1A—Zn—N6—N7	−40.6 (3)	N2B—C3B—C4B—C5B	−179.1 (2)
N1B—Zn—N6—N7	−151.4 (2)	C7B—C3B—C4B—C5B	−0.4 (3)
N3—Zn—N6—N7	82.3 (3)	N2B—C3B—C7B—C6B	178.4 (2)
Zn—N1A—C5A—C4A	−175.6 (2)	C4B—C3B—C7B—C6B	−0.4 (3)
C6A—N1A—C5A—C4A	0.9 (4)	C3A—C4A—C5A—N1A	0.0 (4)
Zn—N1A—C6A—C7A	176.03 (16)	C3B—C4B—C5B—N1B	0.8 (4)
C5A—N1A—C6A—C7A	−0.3 (3)	N1A—C6A—C7A—C3A	−1.1 (3)
Zn—N1B—C5B—C4B	179.65 (19)	N1B—C6B—C7B—C3B	0.9 (4)

Symmetry codes: (i) *x*, −*y*−1/2, *z*+1/2; (ii) −*x*+1, −*y*−1, −*z*+1; (iii) −*x*+1, −*y*, −*z*+1; (iv) −*x*+2, *y*+1/2, −*z*+3/2; (v) −*x*+1, *y*+1/2, −*z*+3/2; (vi) *x*, −*y*−1/2, *z*−1/2; (vii) −*x*+2, −*y*−1, −*z*+2; (viii) −*x*+1, *y*−1/2, −*z*+1/2; (ix) −*x*+2, *y*−1/2, −*z*+3/2; (x) −*x*+1, *y*+1/2, −*z*+1/2; (xi) *x*, −*y*−3/2, *z*−1/2; (xii) −*x*+1, *y*−1/2, −*z*+3/2; (xiii) *x*, −*y*−3/2, *z*+1/2.

### Hydrogen-bond geometry (Å, º)

**Table:**

*D*—H···*A*	*D*—H	H···*A*	*D*···*A*	*D*—H···*A*
C1*A*—H1*A*1···N8^vii^	0.9600	2.5600	3.484 (4)	163.00
C5*A*—H5*A*···N8^ix^	0.9300	2.5900	3.332 (4)	137.00
C7*B*—H7*B*···N3^ii^	0.9300	2.5800	3.367 (4)	143.00
C7*B*—H7*B*···N4^ii^	0.9300	2.5900	3.382 (4)	143.00

Symmetry codes: (ii) −*x*+1, −*y*−1, −*z*+1; (vii) −*x*+2, −*y*−1, −*z*+2; (ix) −*x*+2, *y*−1/2, −*z*+3/2.
